# Co-expression of the Hemagglutinin and Neuraminidase by Heterologous Newcastle Disease Virus Vectors Protected Chickens against H5 Clade 2.3.4.4 HPAI Viruses

**DOI:** 10.1038/s41598-018-35337-z

**Published:** 2018-11-15

**Authors:** Yeonwoo Cho, Barisha Lamichhane, Abdou Nagy, Ishita Roy Chowdhury, Siba K. Samal, Shin-Hee Kim

**Affiliations:** VA-MD Regional College of Veterinary Medicine, University of Maryland, College Park, MD 20742 USA

## Abstract

Avian influenza remains an important zoonotic disease with a significant global impact. The spread of H5 highly pathogenic avian influenza (HPAI) viruses (clade 2.3.4.4) by migratory birds has caused outbreaks in wide geographic regions (Asia, Europe, and North America) with great economic losses during 2014–2015. Efficient vaccines and vaccination approaches are needed to enhance protective immunity against HPAI viruses. Although several vaccination strategies have been developed, none has been satisfactory. Our strategy has been to use avirulent vaccine strain of Newcastle disease virus (NDV) as a vaccine vector for HPAI viruses. For poultry vaccination, we previously generated a new platform of chimeric NDV vector to overcome preexisting maternal antibodies to NDV in poultry. In this study, we have generated vaccine candidates targeting H5 clade 2.3.4.4 HPAI viruses by using our chimeric NDV and conventional NDV strain LaSota vectors for a heterologous prime-boost immunization approach. Co-expression of the HA and NA proteins by our vaccine vectors induced enhanced HPAI virus specific immune responses in specific-pathogen free and broiler chickens prior to challenge. Further, these vaccine candidates efficiently protected broiler chickens from mortality, clinical signs, and shedding of homologous and heterologous H5 HPAI viruses and highly virulent NDV, thus providing a dual vaccination approach in the field.

## Introduction

Avian influenza virus (AIV) belongs to the family *Orthomyxoviridae* and the genus *Influenzavirus* A^1^. The virus has a negative-sense, single-stranded and segmented RNA genome and contains eight gene segments encoding at least 10 proteins: polymerase basic 1 (PB1), PB2, polymerase acid (PA), hemagglutinin (HA), nucleoprotein (NP), neuraminidase (NA), matrix 1 (M1), M2, nonstructural 1 (NS1) and 2 (NS2). The HA and NA proteins are surface glycoproteins and important for virus infectivity. The HA protein is responsible for virus attachment to the host cell and the major target of the humoral immune response. The NA protein plays a role in release and spread of progeny virions by removing sialic acid from glycoproteins.

HPAI virus is an economically-important pathogen of poultry worldwide. Wild aquatic birds are the reservoir of the virus and play an important role in transmitting the virus into susceptible poultry. The outbreaks involving H5 and H7 subtypes of HPAI viruses have resulted in lethal infections in poultry, thus affecting poultry production and trade^2^. In addition, occasional transmission of these viruses to humans has caused great concerns for public health and potential emergence of a new influenza A virus pandemic^3^. Specifically, the cumulated number of confirmed human cases of H5N1 and Asian H7N9 infection reported to WHO to date is 860 with 454 fatal cases (53% mortality) and 1,625 with 623 deaths, respectively^4,5^.

In the past 20 years, the number of HPAI virus outbreaks has increased, and the goose/Guangdong (Gs/GD) H5N1 and Mexican H7N3 lineages of viruses have become endemic in poultry^6^. Continuous circulation of HPAIV has led to a reassortant H5N8 virus (clade 2.3.4.4) in which with the HA gene segment is from an H5N1 HPAI virus and other gene segments are from several other AI viruses circulating in eastern China^7^. Since 2014, H5N8 viruses have spread rapidly via migratory wild aquatic birds and have evolved through reassortment with prevailing local low pathogenic avian influenza (LPAI) viruses. The H5N8 virus and its reassortant viruses have caused outbreaks in wide geographic regions (Asia, Europe, and North America) during 2014–2015.

In the U.S., H5N8 HPAI virus was first detected in wild waterfowl in the Pacific Northwest in 2014^8,9^. Subsequently, the U.S. experienced an unprecedented outbreak of H5 HPAI virus with detections of the virus in wild waterfowl and backyard and commercial poultry flocks throughout the Northwestern and upper Midwestern states across the Pacific, Central, and Mississippi wild bird flyways^10,11^. Reassortment events of H5N8 virus with LPAI viruses further led to the divergence of H5 viruses into distinct subtypes, including H5N1, H5N2, and reassortant H5N8^9,12,13^. H5N2 virus was detected in the Midwest in 2015, causing a devastating outbreak in commercial poultry with an estimated $3.3 billion in economic losses^11,12,14^.

Vaccination of poultry against HPAI viruses could play an important role in reducing virus shedding and raising the threshold for infection and transmission to other birds and humans^15^. However, rapid change in the antigenicity of H5 HPAI virus has been a challenge for efficient control of HPAI virus infection. Antigen matching between the vaccine and the currently circulating field strains is a critical factor in AI virus vaccine efficacy^16–19^. Currently available inactivated vaccines provide suboptimal protection of poultry and require labor intensive administration via the intramuscular route. Inactivated vaccines can be effective generally when the birds are immunologically mature (>3-week-old ages)^20^. Further, administration of prime-and-boost vaccination rounds is challenging with numerous birds in industrial settings and rapid turnover rates of poultry populations^18^. Live virus-based vectored vaccines can be alternative platforms for AI virus vaccines. However, preexisting maternal antibody to the vector can reduce the efficacy of the vaccine in the field. To overcome this limitation, we previously developed an antigenically distinct chimeric NDV vector expressing the protective antigen of H5N1 HPAI (A/Vietnam/1203/2004)^21^. This chimeric NDV vector was generated by replacing the ectodomains of F and HN proteins with those of serologically distinct avian paramyxovirus serotype-2 (APMV-2). Our study demonstrated that a heterologous prime-boost immunization approach using our chimeric NDV and conventional NDV (LaSota) vectored vaccines efficiently protected commercial chickens against HPAI virus challenge^22^. In this study, we have applied our heterologous prime and boost vaccination strategy for efficient control of a novel H5 virus (clade 2.3.4.4) infection in broiler chickens.

## Results

### Generation of NDV vectored vaccine candidates

**Figure 1:**
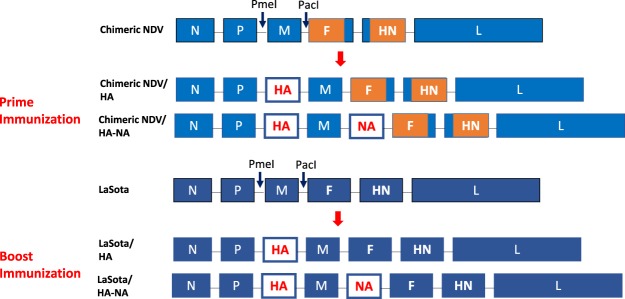
Generation of chimeric NDV and LaSota vectored vaccine viruses (**A**) The HA and NA genes were placed between the P and M genes and between the M and F genes, respectively. Ectodomains of the F and HN genes derived from APMV-2 are shown as orange rectangle.

In this study, our target protective antigens of the HA and NA proteins were from A/Northern Pintail/WA/40964/2014 H5N2, since the outbreak of H5N2 has caused devastating impact in poultry industry^12^. NDV vectored vaccine candidates were constructed by placing codon optimized HA and NA genes between the P and M and the M and F genes, respectively, in the full-length antigenomic cDNAs of chimeric NDV and NDV strain LaSota (Fig. 1). The HA gene was modified to contain the cleavage site of LPAI virus. The generated vaccine candidates were chimeric NDV/HA and chimeric NDV/HA-NA for prime and LaSota/HA and LaSota/HA-NA for boost immunizations. After recovery of the vaccine viruses, the presence of the HA and NA genes without any mutations were confirmed by sequence analysis (data not shown).

**Figure 2:**
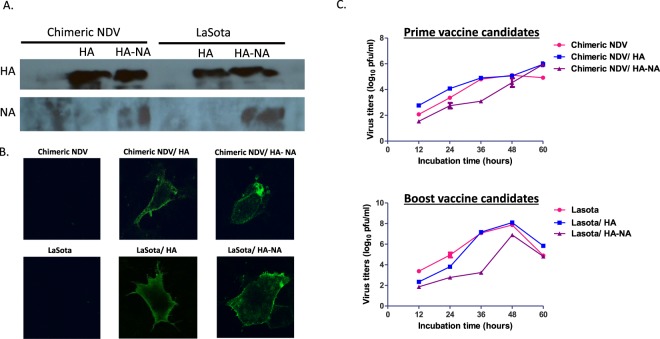
*In vitro* characterization of vaccine candidates. (**A**) Expression of H5N2 HA and NA proteins by NDV vectors were analyzed by Western blot. (**B**) Surface expression of the HA protein was analyzed by confocal microscopy. DF1 cells were infected with each virus at MOI 1, fixed with paraformaldehyde at 24 h post-infection and labeled with anti-HA antibody (chicken polyclonal antiserum to H5N2, Charles River, Norwich, CT) followed by fluorescein isothiocyanate conjugated goat anti-chicken antibody. (**C**) The growth kinetics was determined by infecting DF1 cells with each virus at an MOI of 0.01.

We evaluated the expression of the HA protein by NDV vectors using Western blot analysis and its surface expression by confocal analysis (Fig. 2A,B). We further confirmed the expression of the NA protein by chimeric NDV/HA-NA and LaSota/HA-NA (Fig. 2A). In general, co-expression of the HA and NA proteins did not affect the expression levels of the HA protein (major protective antigen) by our vaccine candidates (Fig. 2A).

We further evaluated the effect of the insertion of HA and NA genes into the genome of NDV vectors on the replication of vaccine candidates in DF1 (Fig. 2C). In prime vaccine group, chimeric NDV/HA replicated efficiently compared to parental chimeric NDV, whereas chimeric NDV/HA-NA did not replicate efficiently until 48 hr post-infection (hpi) (p < 0.05). In boost vaccine group, vaccine candidates did not replicate efficiently compared to LaSota until 24 hpi (p < 0.05). LaSota/HA reached a similar level of titer to that of LaSota in 36 hpi, whereas LaSota/HA-NA did not show a comparable level of replication to that of LaSota (p < 0.05), indicating that co-expression of the HA and NA affected replication of LaSota.

Our vaccine candidates were subjected to the standard pathogenicity test, intracerebral pathogenicity index (ICPI) test in 1-day-old specific-pathogen free (SPF) chicks. We confirmed that the expression of the HA and NA proteins did not affect the avirulent nature of the two NDV vectors. All infected chickens were healthy during the 8 days of infection period (ICPI value of 0.00), thus indicating that they are safe for vaccination of chickens (data not shown).

### Immunogenicity and protective efficacy of chimeric NDV vectored vaccines in SPF chickens

Protective efficacy of vaccine candidates was evaluated by the intranasal immunization of 1-day-old SPF chickens with prime and boost immunization. Our vaccination strategy was to simultaneously express the HA protein as a major antigen and the NA protein as a minor antigen by taking advantage of the nature of polar gradient of NDV transcription^23^. Two groups of divided chickens (20 chickens for each group) were immunized with chimeric NDV/HA and chimeric NDV/HA-NA, respectively (Table 1). For boosting, each group of prime immunized chickens were subdivided into two groups (10 chickens for each group) and immunized with LaSota/HA and LaSota/HA-NA, respectively.

**Table 1:** Immunization of 1-day-old chicks by heterologous prime and boost immunization.

Group	1-day-old chicks		Prime immunization	Boost Immunization
	No.	Route		
1	10	Intranasal	Chimeric NDV/HA	LaSota/HA
2	10	Intranasal	Chimeric NDV/HA	LaSota/HA-NA
3	10	Intranasal	Chimeric NDV/HA-NA	LaSota/HA
4	10	Intranasal	Chimeric NDV/HA-NA	LaSota/HA-NA
Control	4	−	Uninoculated	Uninoculated

**Figure 3:**
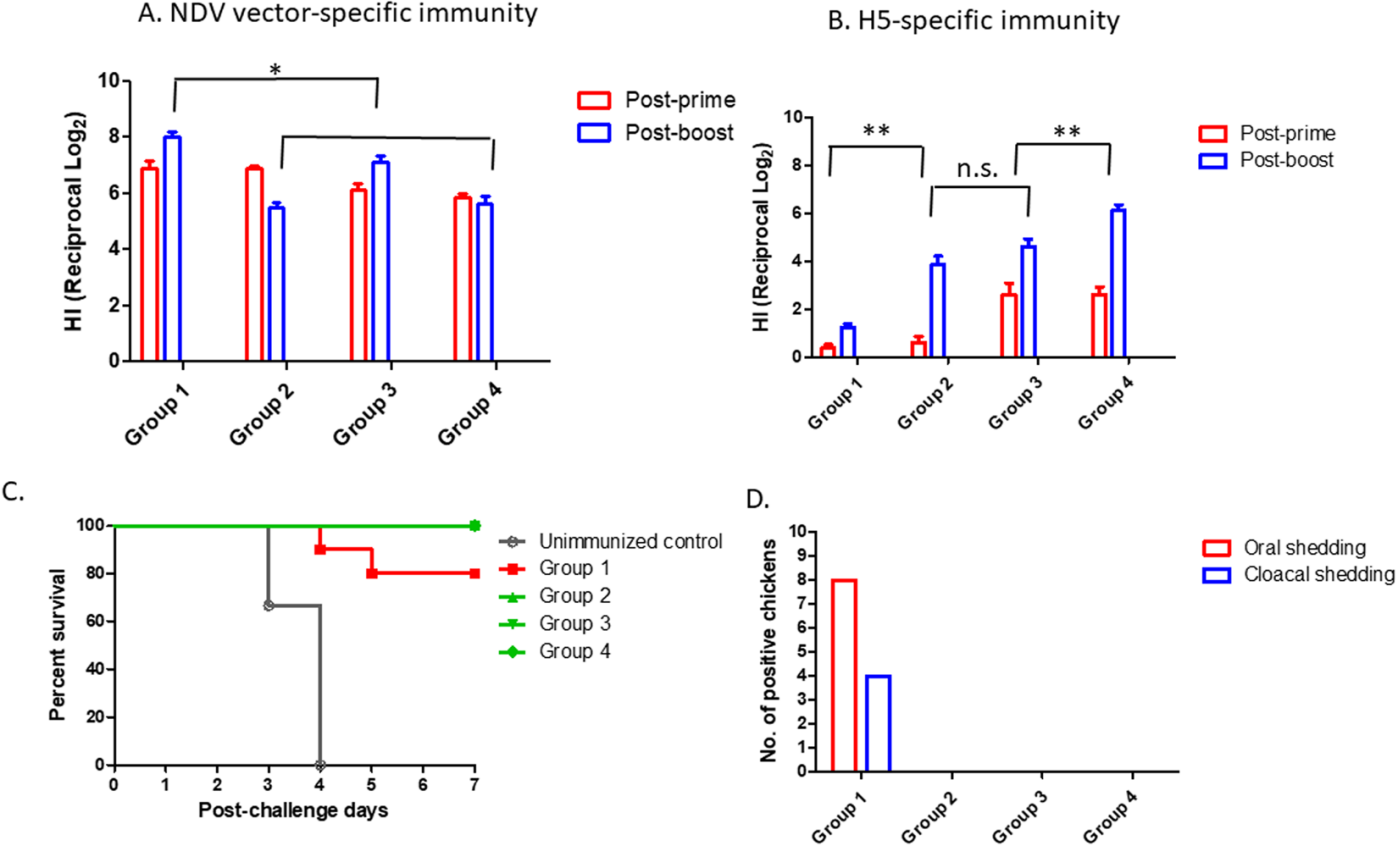
Protective efficacy of NDV vectored vaccines in SPF chickens. SPF chickens were intranasally immunized with chimeric NDV/HA (Groups 1 and 2) or chimeric NDV/HA-NA (Groups 3 and 4). After 2 weeks, the chickens were boosted intranasally with LaSota/HA (Groups 1 and 3) or LaSota/HA-NA (Groups 2 and 4). (**A**) Vector-specific antibodies were determined by a hemagglutination inhibition (HI) assay using chimeric NDV (prime) and LaSota (boost). *Significant difference in vector-specific immunity between LaSota/HA groups (Groups 1 and 3) and LaSota/HA-NA groups (Groups 2 and 4) (p < 0.05). (**B**) H5-specific antibodies were determined by a HI assay using H5N2. ns: no significant difference. **Significant difference in H5-specific immunity between Groups 1 and Groups 2 and 3; and between Groups 2 and 3 and Groups 4 (p < 0.05). Each immunization group of chickens were challenged with H5N2 HPAIV. Mortality (**C**) and virus shedding (**D**) in challenged SPF chickens were evaluated. Oral and cloacal swabs were collected from the chickens at 3 days post challenge, and shedding of the challenge virus was determined by inoculating clarified swab samples into 10-day-old SPF embryonated chicken eggs and conducting HA assay.

Prior to the challenge, we determined the induction of NDV vector- and H5-specific immune responses (Fig. 3A,B). Prime immunization of chickens with chimeric NDV/HA (Groups 1 and 2) and chimeric NDV/HA-NA (Groups 3 and 4) induced similar levels of vector-specific immunity in chickens (Fig. 3A). In contrast, we found significantly higher levels of vector-specific immunity in LaSota/HA groups (Groups 1 and 3) than LaSota/HA-NA groups (Groups 2 and 4), similar to the pattern of *in vitro* replication (Fig. 2C). In H5-specific immunity, we found significantly higher levels of HI titers in chickens prime immunized with chimeric NDV/HA-NA than chimeric NDV/HA (Fig. 3B). Prior to challenge, we found significantly highest level of HI titers in chickens immunized with chimeric NDV/HA-NA and LaSota/HA-NA and the lowest level of HI titers in chickens with chimeric NDV/HA and LaSota/HA (p < 0.05), suggesting that co-expression of the HA and NA is critical for enhancing the protective immunity of NDV vectored vaccines.

Challenge with homologous H5N2 HPAIV resulted in 100% mortality of unimmunized SPF chickens. We found the mortality of infected chickens between 3 and 4 days post-challenge (dpc). In chimeric NDV/HA primed chickens, the mortality (20%) was detected in the LaSota/HA boosted group (Fig. 3C). Shedding of challenge virus was detected in these chickens (8/10 in oral swab, 4/10 in cloacal swab). In contrast, boosting of the chickens with LaSota/HA-NA in the same prime group protected SPF chickens against mortality, clinical signs, and shedding of challenge virus indicating the important role of simultaneous expression of the HA and NA protein in protective immunity (Fig. 3C,D). Prime of chickens with chimeric NDV/HA-NA also greatly improved protective efficacy of vaccination. All the chickens boost with LaSota/HA or LaSota/HA-NA survived up to 7 dpc, and shedding of challenge virus was not detected in the chickens. Our results showed that balanced expression of the HA and NA proteins by NDV vectors were critical in protecting chickens against a lethal dose of H5N2. Therefore, this study enabled us to identify the best strategy by immunization of chickens with chimeric NDV/HA-NA followed by LaSota/HA-NA.

### Efficient protection of broiler chickens against H5 HPAI viruses

**Figure 4:**
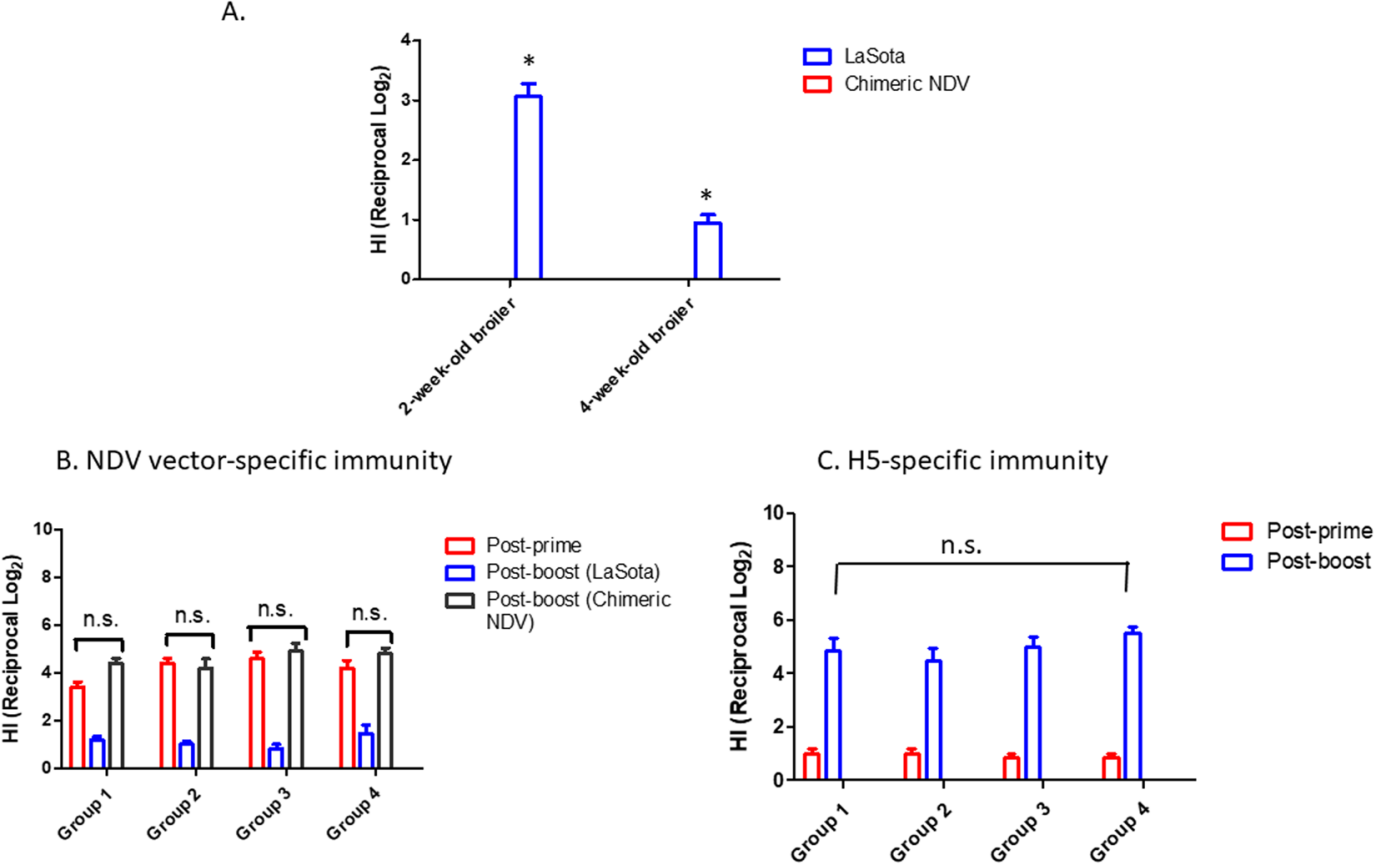
Immunogenicity of NDV vectored vaccines in broiler chickens. Each group of broiler chickens (one-day-old) were intranasally immunized with chimeric NDV/HA-NA and then boost immunized with LaSota/HA-NA intranasally at 2 weeks post-immunization. Serum samples were collected prior to boost and challenge. Vector-specific immunity in broiler chickens was evaluated by a hemagglutination inhibition (HI) assay using chimeric NDV and LaSota (**A**). Virus-specific antibodies were determined by a HI assay using chimeric NDV (prime) and LaSota and chimeric NDV (boost) (**B**) and H5N2 (**C**). n.s.: no significant difference. *Significant difference in LaSota-specific immunity between 2-week-old and 4-week-old broiler chickens (p < 0.05).

We verified the protective efficacy of vaccination strategy in 1-day-old broiler chickens obtained from a commercial hatchery. Since the levels of immunity to NDV broiler chickens varies batch-to-batch, we first determined the changes in NDV vector specific antibody titers in unimmunized chickens (n = 16) (Fig. 4A). We were able to detect LaSota-specific antibody titers in 2-week-old chickens prior to boost immunization, but its presence was diminished in 4-week-old chickens. In contrast, we were unable to detect chimeric NDV-specific antibody in any ages of chickens, suggesting that chimeric NDV could be a good vector for prime immunization.

Prior to the boosting and challenge, we evaluated the induction of NDV vector and HPAI virus specific-antibody titers in immunized chickens. Chimeric NDV/HA-NA induced vector specific-antibody titers in 2-week-old chickens (Fig. 4B). We further confirmed a lasting immune response to chimeric NDV-specific antibody in 4-week-old broiler chickens. However, boosting with LaSota vector did not induce high titers of LaSota-specific antibody in 4-week-old chickens (Fig. 4B). LaSota/HA-NA induced lower HI serum titers in broiler chickens than in SPF chickens (p < 0.05), probably due to the interference of the pre-existing vector immunity in broiler chickens (Fig. 4A). In H5-specific antibody responses, the detected HI titers in chickens were negligible prior to boost. However, we found significantly enhanced HI serum titers in broiler chickens at 2 weeks post-boosting (Fig. 4C). This could be due to the synergistic effects of lasting immunity with chimeric NDV/HA-NA vaccine and boost immunity with LaSota/HA-NA vaccine on protective efficacy.

**Figure 5:**
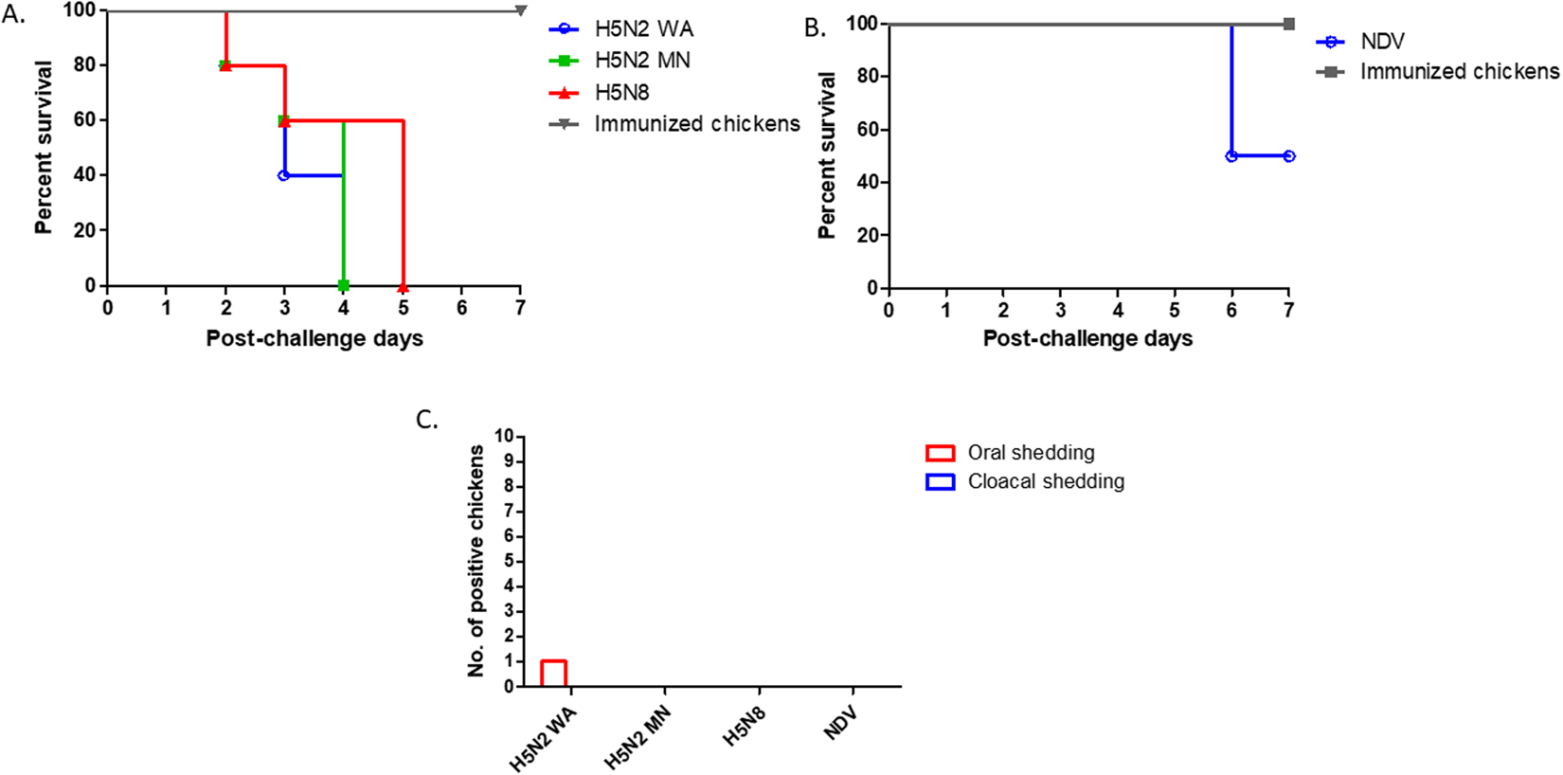
Protective efficacy of NDV vectored vaccines in broiler chickens. Prime-boost immunization groups of broiler chickens were challenged intranasally with H5N2 WA, H5N2 MN, H5N8 or NDV GB Texas. Mortality (**A**,**B**) and shedding of challenge virus (**C**) in broiler chickens were evaluated. Mortality (**A**,**B**) and virus shedding (**C**) in challenged broiler chickens were evaluated. Oral and cloacal swabs were collected from the chickens at 3 days post challenge, and shedding of the challenge virus was determined by inoculating clarified swab samples into 10-day-old SPF embryonated chicken eggs and conducting HA assay.

The protective efficacy of our prime-boost vaccination in broiler chickens was further evaluated by challenging the chickens with homologous and heterologous strains of H5N2 (WA and MN strains, respectively) and H5N8 HPAI viruses (Fig. 5A). We chose to include H5N2 and H5N8 viruses for our challenge study to evaluate whether our vaccine candidates can provide protective immunity to various H5 clade 2.3.4.4 viruses. In unimmunized chickens, we found 100% mortality of chickens challenged with H5N2 viruses at 4 dpc and H5N8 at 5 dpc, respectively. However, all the immunized chickens survived up to 7 dpc. Shedding of challenge virus was detected in one group of chickens challenged with homologous strain of H5N2 (1/10 in oral swab, 0/10 in cloacal swab) at 3 dpc. Challenge with H5N8 also showed that our vaccine candidates with a heterologous NA subtype can protect broiler chickens from mortality and virus shedding. The NA protein targeting vaccines are known to induce a broad protective immunity^24^.

For the purpose of dual vaccination in the field, one group of immunized chickens was challenged with NDV strain GB Texas (GBT) (Fig. 5B). Interestingly, we found only 50% of mortality in unimmunized chickens after a lethal dose of challenge used for SPF chicken experiment. This partial protection of chickens would be correlated with LaSota specific HI titers in unimmunized chickens (Fig. 4A). However, all the immunized chickens were completely protected against mortality and shedding of challenge virus, suggesting that boost immunization with LaSota/HA-NA enhanced protective immunity to a highly virulent NDV.

## Discussion

NDV is an ideal vector for development of HPAI vaccine by inducing local and systemic immune responses at the respiratory tract^3,25^. In the laboratory setting, NDV vectored vaccines have shown to be effective in protecting SPF chickens against HPAI virus infection^15,26–31^. However, vectored vaccines based on NDV, fowlpox virus and turkey herpesvirus have been licensed for AI virus vaccination with limited use (<5% of usage) in China and Mexico^26,32,33^. Despite the advantage of mass vaccination, maternal antibodies to these vaccine vectors have hampered the efficacy of vaccines for early protection of poultry in the field. We also found discrepancy in our vaccine trials between SPF and broiler chickens^22^. Therefore, our study emphasizes on the use of an alternative NDV vaccine vector to overcome maternal antibody in poults and to provide an early and a long-lasting immunity in commercial poultry.

We previously demonstrated potential use of our heterologous prime-boost immunization approach for protection of broiler chickens against A/Vietnam/1203/2004 H5N1 (clade 1). Homologous prime and boost immunization of broiler chickens with LaSota/HA did not provide complete protection against mortality and shedding of challenge virus^22^. Therefore, chickens were not immunized subsequently with homologous vector in this study. However, we intended to improve our boost vaccination strategy by co-expressing the HA and NA proteins by LaSota vector. We found the induction of enhanced levels of HA-specific antibodies in the groups of LaSota/HA-NA immunized chickens (Fig. 3B). Consequently, this vaccination approach provided complete protection of prime immunized SPF chickens even with chimeric NDV/HA (Fig. 3C,D). Most studies have used the HA protein as a protective antigen for NDV vectored vaccines^27^. Our study suggests that co-expression of the HA and NA proteins by NDV vectors can be a novel strategy in enhancing protective efficacy of HPAI virus vaccines in the field. This can be used to design NDV vectored vaccines against other emerging influenza viruses, since the NA antibody has shown to play a role in reducing clinical signs and shedding^34^.

Our previous serological analysis with broiler chickens indicated a lack of pre-existing immunity against our chimeric NDV in the field, supporting that our chimeric NDV can be an effective vector for early vaccination of poultry. The batch of broiler chickens used in this study also showed the lack of pre-existing immunity against our chimeric NDV in broiler chickens (Fig. 4A). One-day-old chicks in a commercial hatchery had received a routine vaccination, including ND vaccine. We have found a lasting LaSota-specific antibody in 2-week-old chickens, and subsequently, a partial protection of unimmunized chickens (4-week-old age) after challenge with a highly virulent NDV strain GBT. This might also attribute to induction of low levels of LaSota specific antibody titers in the boosted chickens with LaSota vector. However, we detected enhanced titers of H5-specific antibodies (>2^4^ HA unit) in those chickens and protection of chickens against H5 viruses (Fig. 4C). Therefore, early protection of chickens against HPAI viruses can be challenging without overcoming the NDV vector immunity in the field.

To ensure the protection of broiler chickens against H5 clade 2.3.4.4 HPAI viruses, we used three challenge viruses: two strains of H5N2 (Northern Pintail and turkey isolates) and H5N8 (a gyrfalcon isolate). Amino acid sequence of the HA protein in our vaccine candidates has 96–99% of identity with those of H5 clade 2.3.4.4 viruses, including Asian strains. This indicates that our vaccine candidates can have a broad application for the vaccination against H5 clade 2.3.4.4 viruses circulating in various regions. In unimmunized chickens, the challenge viruses induced similar patterns of clinical signs and mortality. The viruses had longer incubation periods than A/Vietnam/1203/2004 H5N1 shown in our previous challenge study (100% mortality of chickens at 2 dpc)^21,22^. Interestingly, we also found higher levels of challenge virus shedding in sequentially immunized chickens with chimeric NDV/HA and LaSota/HA (Fig. 3D) compared to our previous study. These H5N2 and H5N8 HPAI viruses are known to be adapted to waterfowl, suggesting that they may not have been adapted to gallinaceous species^8^. Prolonged incubation periods can make it difficult in monitoring AIV infection in domestic farms, since the early detection of HPAI viruses is critical for rapid control and eradication in the U.S. and other countries^35^. Vaccination was not implemented in the 2014–2015 U.S. outbreak of these H5 HPAI viruses; however, the outline of an emergency vaccine bank and vaccination policy for use in the future may be strategic to shorten the time for development and approval for vaccines^20^. In fact, the 2014–15 outbreak demonstrated that inefficient culling process and handling of carcasses in large farms or areas of highly concentrated poultry production allowed shedding of viruses into the environment^36^.

Our study provides a safe and effective vaccination strategy using a novel platform of serologically heterologous viral vectors. In general, use of live attenuated vectored vaccines can be practical compared to conventional inactivated AI vaccines (i.e., cost-effectiveness and application of mass vaccination through spray or drinking water). Therefore, our approach has implication with rapid, efficient, and economical immunization of poultry in the field.

## Methods

### Generation and characterization of chimeric NDV vectored vaccine candidates

The coding sequences for the HA and NA genes of H5N2 were synthesized with codon optimization since this enhanced the levels of protein expression in our previous study. Each ORF of HA (1692 nucleotides in length) and NA (1,407 nucleotides in length) genes was flanked by gene-start and gene-end signals of respective NDV vector. In addition, the original polybasic cleavage site sequence of the HA gene (PLRERRRKR) was replaced by that of LPAIV (PQRETR). The HA gene was placed between the P and M genes of each NDV vector (Fig. 1A). Subsequently, the NA gene was individually placed between the M and F genes of the NDV vectors. Infectious viruses were generated using NDV reverse genetics techniques following our standard protocol^37^. After the recovery of the vaccine viruses, the presence of the HA and NA genes was confirmed by RT-PCR and sequence analysis.

Expression of the HA and NA proteins in virus infected chicken embryo fibroblast cell line (DF1) was confirmed by Western blot analysis. Surface expression of the HA protein by NDV vectors was confirmed by confocal microscopy. The efficiency of *in vitro* replication of our vaccine candidates was determined in virus-infected DF1 cells in duplicate^21^. Virus titers in the collected supernatants were quantified in DF1 cells. The pathogenicity of these vaccine candidates was determined by intracerebral pathogenicity index (ICPI) test in 1-day-old chicks^38^. All the animal experiments were conducted following the guidelines and approval of the Animal Care and Use Committee (IACUC) and Institutional Biosecurity Committee (IBC), University of Maryland.

### Immunogenicity and protective efficacy of the chimeric NDV vectored vaccines in SPF and broiler chickens

The protective efficacy of our vaccine candidates was evaluated by immunizing 1-day-old SPF chicks (Table 1). Forty of chickens were divided into two groups for prime immunization with chimeric NDV/HA (groups 1 and 2) and chimeric NDV/HA-NA (groups 3 and 4) by the intranasal route (100 µl each, 10^6^ pfu/ml). One group of chickens remained uninfected as a control group. After 2 weeks, each group of prime immunized chickens were subdivided into two groups (10 chickens for each group) and further boosted with LaSota/HA (groups 1 and 3) and LaSota/HA-NA (groups 2 and 4), respectively (200 µl each, 10^6^ pfu/ml). Prior to boost and challenge experiments, serum samples were collected to monitor the immune responses in the chickens. The antibody titers in serum samples were determined by HI assay using NDV strain LaSota, chimeric NDV or H5N2 as an individual antigen to evaluate the induction of vector-specific and H5-specific antibody responses^21^. At 2 weeks post-boost, challenge experiment was conducted in our enhanced biosafety level-3 facility. Each group of chickens was intranasally challenged with a lethal dose of H5N2 (A/Northern Pintail/WA/40964/2014, 10^6^ pfu/ml). All the challenged chickens were evaluated on a daily basis for mortality and clinical signs up to 7 dpc. To monitor the shedding of the challenge virus, oral and cloacal swabs were collected at 3 dpc, inoculated into 9-day-old SPF embryonated chicken eggs, and confirmed by HA assay using chicken erythrocytes.

The protective efficacy of our best vaccine candidates was further evaluated by prime-boost immunization of 1-day-old broiler chickens (Cornish Cross, Amick Farms Hurlock Hatchery, Hurlock, MD) following the protocol described for SPF chickens. Broiler chickens were prime immunized with chimeric NDV/HA-NA and boosted with LaSota/HA-NA. One group of broiler chickens (n = 16) remained uninfected to monitor NDV vector-specific immune responses. At 2 weeks post-infection, broiler chickens were subdivided into four groups (10 chickens for each group) and intranasally challenged with a lethal dose of H5N2 (A/Northern Pintail/WA/40964/2014 and A/turkey/Minnesota/9845-4/2015), H5N8 (A/gyrfalcon/Washington/41088-6/2014) (200 µl of 10^6^ pfu/ml), and highly virulent NDV strain GB Texas (200 µl of 10^4^ pfu/ml), respectively, in our ABSL-3 facility.

### Statistical analysis

Statistically significant differences in serological analysis of different immunized chicken groups were evaluated by one-way analysis of variance (ANOVA) using the Turkey’s multiple comparison test. The survival rate was compared using the log-rank test and chi-square statistics. All the results were analyzed by using Prism 5.0 (GraphPad Software Inc., San Diego, CA) with a significance level of P < 0.05.
